# Population-based variance-reduced evolution over stochastic landscapes

**DOI:** 10.1038/s41598-025-18876-0

**Published:** 2025-10-07

**Authors:** Zelin Pei, Xiaoyu He, Yi Pan, Baichun Peng, Wen Chen, Yuren Zhou

**Affiliations:** 1 School of Software Engineering, Sun Yat-Sen University, Guangzhou, 510006 China; 2 Energy Development Research Institute, China Southern Power Grid Company Limited, Guangzhou, China

**Keywords:** Applied mathematics, Computer science

## Abstract

Black-box stochastic optimization involves sampling in both the solution and data spaces. Traditional variance reduction methods mainly designed for reducing the data sampling noise may suffer from slow convergence if the noise in the solution space is poorly handled. In this paper, we present a novel zeroth-order optimization method, termed Population-based Variance-Reduced Evolution (PVRE), which simultaneously mitigates noise in both the solution and data spaces. PVRE uses a normalized-momentum mechanism to guide the search and reduce the noise due to data sampling. A population-based gradient estimation scheme, a well-established evolutionary optimization technique, is incorporated to further reduce noise in the solution space. We show that PVRE exhibits the convergence properties of theory-backed optimization algorithms and the adaptability of evolutionary algorithms. In particular, PVRE achieves the best-known function evaluation complexity of $\mathscr {O}(n\epsilon ^{-3})$ for finding an $\epsilon$-accurate first-order optimal solution, up to a logarithmic factor, with any initial step-size. We assess the performance of PVRE through numerical experiments on benchmark problems as well as a real-world task involving adversarial attacks against neural image classifiers.

## Introduction

We consider the unconstrained optimization problem

1 $$\begin{aligned} \min _{x \in \mathbb {R}^n} f(x) = \mathbb {E}_{\xi \sim \mathscr {D}}[F(x;\xi )], \end{aligned}$$

where $x \in \mathbb {R}^n$ is the solution to be found, $\mathscr {D}$ is the data distribution, and $\xi$ denotes a random data sample. As in most real-world scenarios, we assume that computing the expectation is intractable due to the large number of data samples or the lack of prior knowledge of the distribution.

Problems in the form of (1) are usually solved by stochastic methods^1^ in which the objective function *f*(*x*) is replaced by some ease-to-compute estimates $F(x;\xi )$. The stochastic gradient descent (SGD)^2^ and its modern variants^3^ are representative of these methods. They typically draw a random sample $\xi$ from $\mathscr {D}$ at each iteration and evaluate the stochastic gradient $\nabla F(x;\xi )$ as an estimate of the true gradient $\nabla f(x)$. First-order information (i.e., the gradient oracle) plays a vital role in these methods since it gives the descent directions for exploring the solution space. These methods are not applicable, however, when gradients are not accessible. This situation, which we call the black-box setting, arises either when the gradient is nonexistent (e.g., the objective value is obtained from physical simulations), or when access to the intrinsic structure of the objective is restricted for security reasons^4^. Even in the ideal situation where automatic differentiation techniques^5^ are available, the memory overheads and the strict software requirements can become a bottleneck^6^ and limit the usability of gradient-based methods.

This work considers zeroth-order approaches to the problem (1) in the sense that we only have access to the stochastic objective value $F(x;\xi )$. Gaussian smoothing^7^ is one of the most popular techniques for estimating gradients in zeroth-order optimization. The key idea is first to sample a Gaussian vector in the solution space and then output the finite difference along this vector as a stochastic gradient estimator. Gaussian smoothing is computationally efficient, theoretically sound, and easily integrated into most first-order methods^8^. However, Gaussian smoothing in the stochastic setting involves sampling in both data and solution spaces, inducing strong gradient noise which can slow down the convergence. Although various variance reduction methods^9^ have been developed to address the noise issue, they are mostly restricted to the data sampling procedure, and their effectiveness can be limited due to the noise in the solution space. They also need extensive hyperparameter tuning or periodic gradient evaluations on mega-batches^10^.

Evolutionary algorithms (EAs)^11,12^ are a family of meta-heuristics that act as a powerful alternative to Gaussian smoothing-based gradient methods for zeroth-order optimization. Compared to traditional gradient-based methods relying on single-point iterations, EAs evolve a population of solutions and possess inherent tolerance to the noise in the solution space^13^. In addition, modern EAs such as evolution strategies^14,15^ are valued for their strong adaptation ability and robustness to complex landscapes^16^. These properties make them very suitable for handling complicated problems such as reinforcement learning^17^, combinatorial optimization^18^, and neural architecture search^19^. The limitation of EAs, however, is that they have no theoretical guarantees unless on a small number of benchmark problems.

In this paper, we combine the theoretical soundness of Gaussian smoothing-based gradient algorithms and the adaptability of EAs for tackling zeroth-order stochastic problems (1). The main novelty of our method lies in two aspects: 1) unlike existing variance-reduction approaches that primarily target data sampling noise in the data space, our method *simultaneously reduces both data sampling noise and solution space sampling noise* by integrating a recursive momentum rule with a population-based search strategy; 2) it introduces a normalization-based step-size adaptation mechanism that *ensures global and adaptive convergence with any initial step-size settings*, eliminating the need for delicate hyperparameter tuning. We demonstrate that the proposed Population-based Variance-Reduction (PVRE) algorithm attains an $\epsilon$-accurate first-order optimal solution (i.e., a solution with gradient norm below $\epsilon$) within $\tilde{\mathscr {O}}(n\epsilon ^{-3})$ function evaluations, matching the best-known complexity bounds^10,20^.

**Notations** $\Vert \cdot \Vert$ denotes $\ell _2$ norm. $\mathscr {N}$ denotes the *n*-dimensional isotropic Gaussian distribution. $\mathbb {E}$ denotes taking expectation. $[\tau ]$ denotes the set $\{1,\ldots ,\tau \}$.

## Preliminaries and assumptions

We review two related techniques, namely the Gaussian smoothing method^7^ for zeroth-order gradient estimation and the STORM method^21^ for data-induced gradient noise reduction. We also list the assumptions required for performance analyses.

### Gaussian smoothing

We start by defining the smoothness of a function, as it impacts the accuracy of Gaussian smoothing-based gradient estimation. The detailed proof is provided in Appendix A.

#### Definition 1

(*Smoothness*) Let *h* be a differentiable function. We say *h* is *L*-smooth if its gradient is *L*-Lipschitz continuous, i.e.,

$$\Vert \nabla h(x) - \nabla h(y)\Vert \le L \Vert x - y\Vert ,\quad \forall x,y,$$

holds for some constant $L \in \mathbb {R}_+$.

The *L*-smoothness of a function *h* implies the following quadratic bounds:

2 $$\begin{aligned} |h(y) - h(x) - \langle \nabla h(x), y - x \rangle |&\le \frac{L}{2}\left\| x - y \right\| ^2, \end{aligned}$$

3 $$\begin{aligned} |h(x + v) - h(x - v) - 2 \langle \nabla h(x), v \rangle |&\le L \Vert v\Vert ^2, \end{aligned}$$

for all *x*, *y*, and *v*.

The Gaussian smoothing of a function at solution *x* is the expected objective value over a Gaussian distribution with mean *x* and a predefined variance:

#### Definition 2

(*Gaussian smooth approximation*) Fix a function $h:\mathbb {R}^n \rightarrow \mathbb {R}$. The Gaussian smooth approximation of *h* is defined as

$$h_\eta (x) = \mathbb {E}_{v \sim \mathscr {N}}[h(x + \eta v)]$$

where $\eta \in \mathbb {R}_+$ is called the smoothing radius.

The approximation above has a nice property that it is differentiable even when the original function is not. Moreover, it has closed-form gradients depending only on objective function values. This property was given in Theorem 2 of the work^7^, and for completeness we restate it below:

#### Proposition 1

(Gradient estimation based on Gaussian smoothing) *Assume a function* $h:\mathbb {R}^n \rightarrow \mathbb {R}$ *is differentiable. Let* $v \in \mathbb {R}^n$ *be a perturbation vector and* $g \in \mathbb {R}^n$ *the finite-difference of* *h* *along* *v* *as*

4 $$\begin{aligned} g = \frac{h(x + \eta v) - h(x - \eta v)}{2 \eta } v, \end{aligned}$$

*where* $\eta \in \mathbb {R}_+$*. Then* *g* *is an unbiased estimator of* $\nabla h_{\eta }(x)$ *if* *v* *is Gaussian distributed and independent of* *x, i.e.,*

$$\mathbb {E}_{v \sim \mathscr {N}}[g] = \nabla h_{\eta }(x).$$

Proposition 1 is critical as it provides a way to compute an unbiased gradient estimator via evaluating only two solutions. In the next section, we will apply Gaussian smoothing to the component function $F(x;\xi )$ in problem (1) to obtain a gradient estimator.

### STORM

STORM, short for STOchastic Recursive Momentum^21^, is a mechanism for reducing the variance due to data sampling in first-order settings. Considering the problem (1) and supposing that the stochastic gradient $\nabla F(x;\xi )$ is available, STORM applies the following descent step

5 $$\begin{aligned} x_{t+1} = x_t - \gamma _t d_t, \end{aligned}$$

with the momentum term $d_t \in \mathbb {R}^n$ defined recursively as

6 $$\begin{aligned} d_t = \underbrace{(1-a_{t-1}) d_{t-1} + a_{t-1} \nabla F(x_t;\xi _t)}_{\text {momentum}} + (1-a_{t-1})\underbrace{(\nabla F(x_t;\xi _t) -\nabla F(x_{t-1};\xi _t))}_{\text {error correction}}, \end{aligned}$$

where $\gamma _t \in \mathbb {R}_+$ is the descent step-size, $a_{t-1} \in (0,1)$ is the momentum learning rate, and $\xi _t$ is a data sample drawn from $\mathscr {D}$. The momentum $d_t$ is intended to capture the deterministic gradient $\nabla f(x_t)$, provided that the component function $F(x;\xi )$ is smooth. One may view $d_t$ as a combination of standard momentum and correction terms. The term $(1-a_{t-1}) d_{t-1} + a_{t-1} \nabla F(x_t;\xi _t)$ acts as a classical heavy-ball momentum^22^ that incorporates stochastic gradient estimations into previous descent step. The term $\nabla F(x_t;\xi _t) - \nabla F(x_{t-1};\xi _t)$ corrects the momentum term via including the gradient difference evaluated with the same data sample, aiming to reduce the variance introduced in previous data sampling. The effectiveness of STORM depends on a prerequisite that the error correction term must approach 0 when the iterations converge, in which case the momentum term acts as a Monte Carlo gradient estimator averaged over *t*.

### Assumptions

We make the following assumptions regarding the problem (1).

#### Assumption 1

(*Boundedness of the objective value*) The objective value is lower bounded by some constant $f_* \in \mathbb {R}$, i.e.,

$$f(x) \ge f_*, \quad \forall x.$$

#### Assumption 2

(*Unbiasedness and boundedness of the data sampling*) The data sampling is unbiased, i.e.,

$$\mathbb {E}_{\xi \sim \mathscr {D}}[\nabla F(x;\xi )] = \nabla f(x), \quad \forall x.$$

In addition, there exists some constant $\sigma \in \mathbb {R}_+$ such that

$$\mathbb {E}_{\xi \sim \mathscr {D}}[ \Vert \nabla F(x;\xi ) - \nabla f(x)\Vert ^2] \le \sigma ^2, \quad \forall x.$$

#### Assumption 3

(*Smoothness of the component function*) The component function $F(x;\xi )$ is *L*-smooth in *x* for all $\xi$.

#### Assumption 4

(*Boundedness of the gradient norm*) The norm of the gradient is upper bounded by some constant $G \in \mathbb {R}_+$, i.e.,

$$\Vert \nabla f(x)\Vert \le G,\quad \forall x.$$

Assumptions 1 and 2 are customary in analyzing stochastic optimization algorithms. Assumption 3 is relatively restrictive, as it necessitates the smoothness of each component function. Nevertheless, it is the key in achieving the $\mathscr {O}(T^{-\frac{1}{3}})$ convergence rate and was typically made in recent studies relying on STORM. Notice that almost all existing works using Gaussian smoothing, to our knowledge, require this assumption to establish convergence theorems, even for getting a slower rate of $\mathscr {O}(T^{-\frac{1}{4}})$^23^. This means, compared to existing Gaussian smoothing based zeroth-order algorithms, we can achieve a speedup in convergence for free. Assumption 4 is used in guaranteeing the adaptability. We note that this is looser than the uniform bound (i.e., $\Vert \nabla F(x;\xi )\Vert \le G$ for all $\xi$) typically used in STORM based methods^21,24^.

Hereinafter we denote the Gaussian smooth approximations of *f* and *F* by $f_{\eta }$ and $F_{\eta }$, respectively, i.e.,

$$f_{\eta }(x) = \mathbb {E}_{v\sim \mathscr {N}}[f(x+\eta v)], \quad F_{\eta }(x;\xi ) = \mathbb {E}_{v\sim \mathscr {N}}[F(x+\eta v;\xi )].$$

## PVRE: Population-based variance-reduced evolution

We present the PVRE method for handling problem (1) in black-box settings. PVRE combines Gaussian smoothing and STORM to reduce the sampling noise in both the data space and the solution space. It also uses a normalized descent rule to achieve adaptability.

### Adaptive descent with normalized momentum

PVRE employs the following normalized descent rule

7 $$\begin{aligned} x_{t+1} = x_t - \gamma _t \frac{d_t}{\Vert d_t\Vert } \end{aligned}$$

where $d_t \in \mathbb {R}^n$ is a descent step. The use of normalization is critical to achieving adaptability. To see this, denote $\epsilon _t$ as the difference between $d_t$ and $\nabla f_{\eta _t}(x_t)$:

8 $$\begin{aligned} \epsilon _t = d_t - \nabla f_{\eta _t}(x_t). \end{aligned}$$

Then we can bound the per-iteration progress using $\epsilon _t$.

#### Lemma 1

(Per-iteration progress) *The detailed proof is provided in Appendix B. Under Assumption* 3*, the iterations* $\{x_t\}$ *generated by (**7**) satisfy*

$$f_{\eta _{t+1}}(x_{t+1}) \le f_{\eta _t}(x_t) - \frac{\gamma _t}{3} \Vert \nabla f_{\eta _t}(x_t) \Vert + \frac{8}{3} \gamma _t \Vert \epsilon _t\Vert + \frac{L}{2} \left( \gamma _t^2 + (\eta _t^2 + \eta _{t+1}^2) n \right) .$$

Lemma 1 states that, once the estimation error $\epsilon _t$ is small, sufficient descent in the smoothed objective $f_{\eta _t}$ can be ensured. Moreover, this property is ensured adaptively in two aspects: 1) it is independent of the data noise (as it does not rely on Assumption 2), and 2) knowledge of the problem-dependent constant *L* is not required, as the *L*-related term in the bound can be made arbitrarily small by decreasing the hyperparameters $\gamma _t, \eta _t$, and $\eta _{t+1}$.

It is now clear that all we need is to make sure $d_t \approx \nabla f_{\eta _t}(x_t)$ so that $\epsilon _t$ can be bounded. We achieve this by using the following update rule:

9 $$\begin{aligned} {\left\{ \begin{array}{ll} d_t = (1-a_{t-1}) d_{t-1} + a_{t-1} g_t + (1-a_{t-1})(g_t - \tilde{g}_{t-1}) \\[6pt] d_0 = g_0 \end{array}\right. } \end{aligned}$$

where $a_{t-1} \in (0,1)$ is a constant, and $g_t, \tilde{g}_{t-1} \in \mathbb {R}^n$ are intended to satisfy

$$\mathbb {E}[g_t] = \nabla f_{\eta _t}(x_t) \text { and } \mathbb {E}[\tilde{g}_{t-1}] = \nabla f_{\eta _{t-1}}(x_{t-1}).$$

Comparing (9) with (6), it is found that the descent step $d_t$ follows the momentum update rule of STORM, with the exception that we replaced the objective function *f* with its Gaussian smoothing. Our idea here is to obtain $g_t$ and $\tilde{g}_{t-1}$ by applying the finite-difference rule (4) to the component function *F* and then inject them into (9). One core difficulty exists, however, in that whereas the correction term in (6) only reduces the noise due to data sampling, applying finite-differences would introduce additionally noise due to solution sampling. Technically speaking, the error correction term may not diminish (i.e., $\Vert g_t -\tilde{g}_{t-1}\Vert \not \rightarrow 0$) even when the iterations converge, which would impact the variance reduction effect. We overcome this by using a population mechanism and a perturbation reuse strategy in gradient estimation.

### Population-based gradient estimation with perturbation reuse

We show how to compute $g_t$ and $\tilde{g}_{t-1}$ in (9). To obtain $g_t$, we generate a set of Gaussian perturbations $\{v_{t,k} \sim \mathscr {N}\}_{k\in [\tau ]}$ where $\tau$ is the population size. We also draw a set of data samples $\{\xi _{t,k} \sim \mathscr {D}\}_{k\in [\tau ]}$. Then, apply the finite-difference rule (4) to *F* with pairs of $\xi _{t,k}$ and $v_{t,k}$ as

10 $$\begin{aligned} g_{t} = \frac{1}{\tau } \sum _{k=1}^{\tau } g_{t,k}, \quad g_{t,k} = \frac{F(x_{t}+\eta _t v_{t,k};\xi _{t,k}) - F(x_t-\eta _t v_{t,k};\xi _{t,k}) }{2\eta _t} v_{t,k}, \end{aligned}$$

where $\eta _t \in \mathbb {R}_+$ is the corresponding smoothing radius.

#### Lemma 2

(Population based variance reduction) *The detailed proof is provided in Appendix B. Suppose Assumptions* *4**and* *2**hold and let*

$$\rho _t = \frac{L^2}{2} \eta _t^2(n+6)^3 + 2(n+4) (G^2+\sigma ^2).$$

*Then, we have*

$$\mathbb {E}\left[ \left\| g_t - \nabla f_{\eta _t}(x_t) \right\| ^2 \right] \le \frac{\rho _t}{\tau }.$$

We now proceed on computing $\tilde{g}_{t-1}$ for estimating $\nabla f_{\eta _{t-1}}(x_{t-1})$. Intuitively, one can draw another population of Gaussian perturbations and perform Gaussian smoothing similarly as in (10). But we propose that a better way is to *reuse the population* $\{v_{t,k}\}_{k\in [\tau ]}$ that has been used for gradient estimation at $x_t$. Specifically, given the population $\{v_{t,k}\}_{k\in [\tau ]}$ and the corresponding data samples $\{\xi _{t,k}\}_{k\in [\tau ]}$ used in (10), we perform gradient estimation at solution $x_{t-1}$ as

11 $$\begin{aligned} \tilde{g}_{t-1} = \frac{1}{\tau } \sum _{k=1}^{\tau } \tilde{g}_{t-1,k}, \quad \tilde{g}_{t-1,k} = \frac{F(x_{t-1}+\eta _{t-1} v_{t,k};\xi _{t,k}) - F(x_{t-1}-\eta _{t-1} v_{t,k};\xi _{t,k}) }{2\eta _{t-1}} v_{t,k}, \end{aligned}$$

where $\eta _{t-1}$ is the smoothing radius that may differ from $\eta _t$.

The perturbation reuse in (11) is the key step of PVRE. At first glance, $\tilde{g}_{t-1}$ is simply an unbiased estimator of $\nabla f_{\eta _{t-1}}(x_{t-1})$, according to Proposition 1. But we notice that the underlying reason lies in that the difference between these two gradient estimators can be upper bounded when they are constructed with the same population of perturbations.

#### Lemma 3

(Boundedness of the correction term) *The detailed proof is provided in Appendix B. Under Assumption* *3**, the gradient estimations given in* *10**and* *11**satisfy*

$$\mathbb {E}\left[ \Vert g_{t,i} - \tilde{g}_{t-1,i}\Vert ^2\right] \le \frac{3L^2(\eta _t^2+\eta _{t-1}^2)}{4}(n+6)^3 + 3(n+4)L^2\gamma _{t-1}^2.$$

Lemma 3 implies that, when $\eta _t,\eta _{t-1}$, and $\gamma _{t-1}$ are sufficiently small, the correction term in (9) (i.e., $g_t - \tilde{g}_{t-1}$) is negligible, and the iterations will converge (i.e., $x_t \approx x_{t-1}$). The momentum $d_t$ then behaves as a Monte Carlo estimator of $\nabla f_{\eta _t}(x_t)$ whose variance reduces over iterations. This is confirmed by the following lemma.

#### Lemma 4

(Boundedness of gradient estimation errors) *The detailed proof is provided in Appendix B. Consider the iterations generated from (**7**) with the settings* *9**to* *11*.

Choose the hyperparameters as

12 $$\begin{aligned} \eta _t = \eta _0 (t+1)^{-2/3}, \quad \gamma _t = \gamma _0 (t+1)^{-2/3}, \quad a_t = (t+1)^{-2/3}, \quad \eta _0 = \frac{\gamma _0}{n+6}. \end{aligned}$$

Under Assumptions 2 to 4, the gradient estimation error defined in (8) can be bounded as

$$\sum _{t=0}^{T-1} \gamma _t \mathbb {E}[\Vert \epsilon _t\Vert ] \le 9 \gamma _0 (\gamma _0 L + G+\sigma )(1+\log T)\sqrt{\frac{n+6}{\tau }}.$$

### Main results

We now present the PVRE method by putting all above details together. The pseudocode is given in Algorithm 1. Below we establish the convergence theorem of the proposed method. The detailed proof is provided in Appendix C.

**Algorithm 1:**
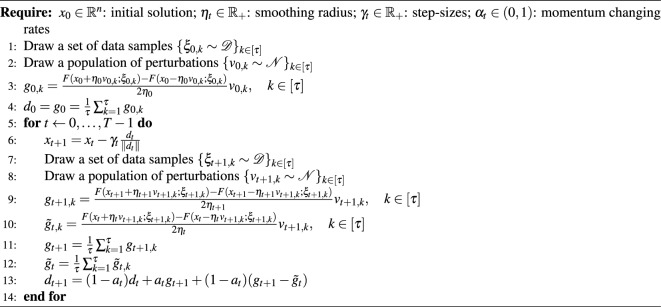
Population-based Variance-Reduced Evolution (PVRE).

#### Theorem 1

*Consider solving problem (**1**) using Algorithm 1 with settings in (**12**). Suppose Assumptions* *4**to* *3**hold. Then the iterations satisfy*

$$\begin{aligned} \frac{1}{T}\sum _{t=0}^{T-1} \mathbb {E}[\Vert \nabla f(x_t) \Vert ] \le 72T^{-\frac{1}{3}}\left( \frac{\Delta _f}{\gamma _0} + (\gamma _0L+ G +\sigma )(1+\log T) \sqrt{\frac{n+6}{\tau }} + \gamma _0L\right) + 2L\sqrt{n+6} \gamma _0 T^{-\frac{2}{3}}, \end{aligned}$$

*where* $$\Delta _f = f(x_0) - f_*$$.

#### Remark

(Function evaluation complexity) The bound given above can be tightened when choosing $\gamma _0 = \Theta \left( \sqrt{\frac{\Delta _f}{L\left( 1+\sqrt{\frac{n+6}{\tau }} \right) }} \right)$, which yields

$$\frac{1}{T}\sum _{t=0}^{T-1} \mathbb {E}[\Vert \nabla f(x_t) \Vert ] = \tilde{\mathscr {O}}\left( \frac{1+\log T}{T^{\frac{1}{3}}} \left( \sqrt{\Delta _f L }\left( 1+ \left( \frac{n}{\tau }\right) ^{\frac{1}{4}} \right) + (G+\sigma )\sqrt{\frac{n}{\tau }} \right) \right)$$

where the $\tilde{\mathscr {O}}$ notation hides the negligible $\log T$ term. Now choose $\tau = \Theta (n)$ and consider $L,\Delta _f,G$, and $\sigma$ as constants. Then PVRE guarantees $\min \limits _{0\le t<T} \mathbb {E}[\Vert \nabla f(x_t)\Vert ] \le \epsilon$ within $T = \tilde{\mathscr {O}}\left( \frac{1}{\epsilon ^3}\right)$ iterations. As $4\tau$ function evaluations are performed at each iteration, we conclude that the function evaluation complexity for PVRE to output an $\epsilon$-accurate first-order optimal solution is $\tilde{\mathscr {O}}\left( \frac{n}{\epsilon ^3}\right)$. This aligns with the best known result given in^25^, up to a $\log T$ factor, which is caused by the diminishing step-size rule. We note that the main advantage of PVRE over existing methods lies in its adaptive convergence, which requires no prior knowledge of the problem landscape characteristics (e.g., *L* and $\sigma$).

## Experiments

We evaluate the performance of PVRE on several benchmark problems and a real-world adversarial attack task. Additional studies on the impact of population size and initial step size are also present.

### Binary classification based benchmark problems

We evaluate PVRE on three binary classification models, including logistic regression (LR), non-convex support vector machine (NSVM), and linear support vector machine (LSVM). The objective function is given by:

$$F(x;\xi ) = loss (x;z,y) = {\left\{ \begin{array}{ll} \log (1+\exp (-y(x^\top z))) & \text {(LR)}\\ 1-\tanh (y(x^\top z)) & \text {(NSVM)}\\ \max \left\{ 0,1-y(x^\top z)\right\} & \text {(LSVM)}\\ \end{array}\right. }$$

where each data sample $\xi$ is a pair of input feature $z \in \mathbb {R}^n$ and the associated label $y \in \{-1,1\}$. LR is the simplest model as it is convex and smooth; we choose it to test the algorithm’s exploitation ability. NSVM is smooth but nonconvex, and we use it to verify the global exploration performance of an algorithm. LSVM deviates from the smoothness assumption, and is used mainly for testing whether an algorithm is robust to irregularities on the landscape.

The data distribution $\mathscr {D}$ consists of *N* data samples, i.e., $\mathscr {D} = \{\xi _1,\dots ,\xi _N\}$, and is constructed using four widely-used benchmark datasets: covtype, gisste, rcv1, and real-sim. Table 1 provides a brief summary of these statistics.

**Table 1:** Statistical summary of the datasets used.

dataset	*n*	*N*
covtype	54	581,012
gisste	5,000	6,000
rcv1	47,236	677,399
real-sim	20,958	72,309

We compare PVRE with five state-of-the-art algorithms, including ZO-SGD^23^, ZO-SignSGD^26^, ZO-AdaGrad^27^, ZO-Adam^28^, and ZO-STORM^21^. Since the original STORM method was not designed for a zeroth-order setting, we construct its zeroth-order variant, referred to as ZO-STORM, by replacing its gradient oracles with a Gaussian smoothing-based gradient estimator. All algorithms use the finite-difference gradient estimator (10) with $\tau = 20$. The minibatch size is set to 1000 for all algorithms. In Gaussian smoothing, we set the initial smoothing radius as $\eta _0 = 10^{-6}$, and adopt a diminishing schedule given by $\eta _t = \eta _0 / (1+t)^{2/3}$. All algorithms are initialized at $x_0 = (0,\dots ,0)^\top$ and employ a diminishing step-size schedule $\gamma _t = \gamma _0 / (1+t)^{2/3}$ throughout the optimization process. The initial step-size $\gamma _0$ is fine-tuned by a grid-search in $\{10^{-4},10^{-3},\dots ,10^4\}$. Every algorithm is run independently 11 times on each dataset. The results from the median run (whose final training loss is the median among the 11 outputs) are reported.

**Fig. 1:**
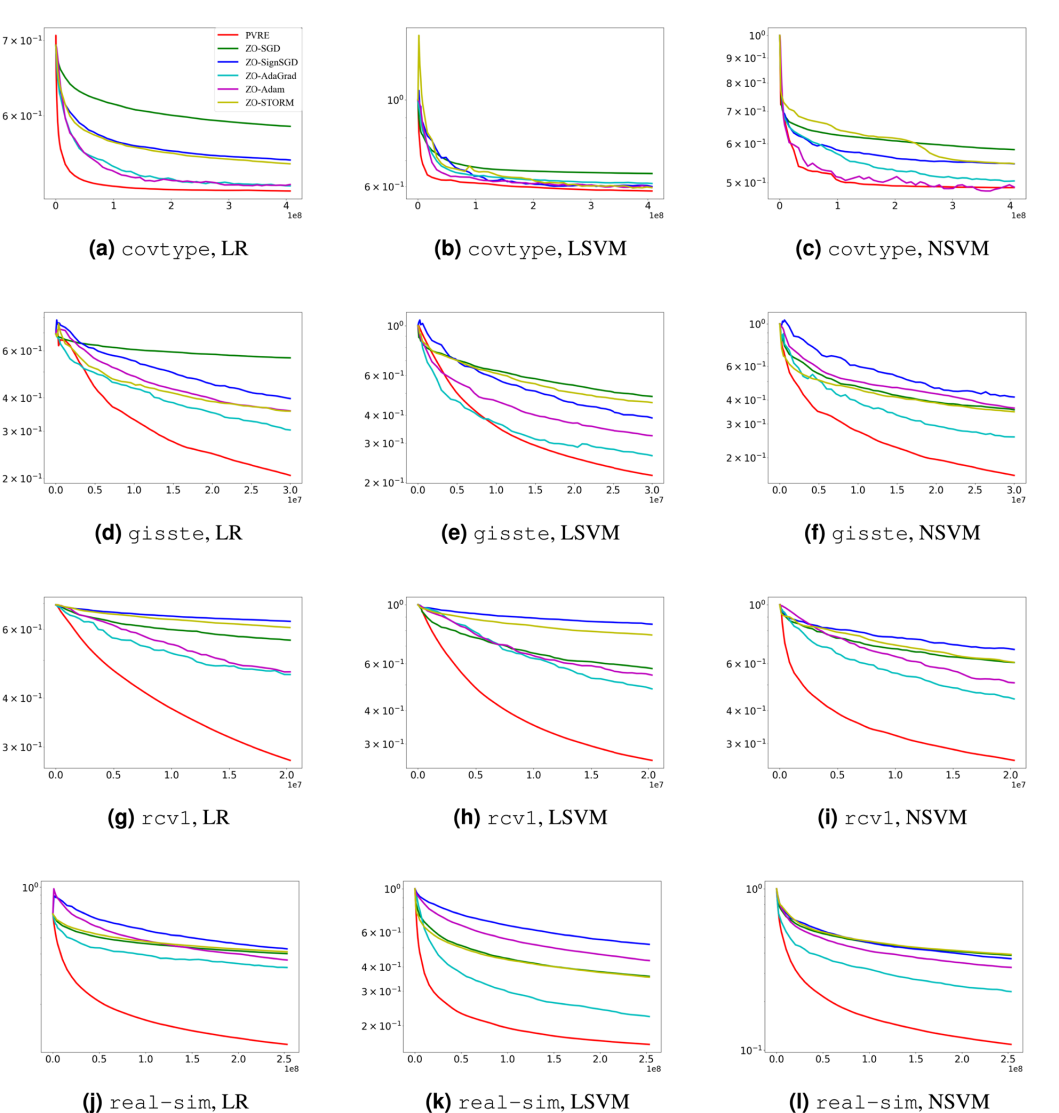
Comparison on benchmark problems. The curve displays the training loss versus the number of function evaluations.

Figure 1 shows the convergence performance of the algorithms. PVRE is clearly the best performer and enjoys faster convergence speed. We also notice that PVRE performs consistently well on NSVM even though this problem does not satisfy the componentwise smoothness assumption. This implies PVRE is robust against irregular landscape features.

### Impact of population size

PVRE uses a population-based search mechanism to guide the optimization process. To evaluate the impact of this mechanism, we conduct experiments to examine how the population size parameter $\tau$ affects performance. Specifically, we test the LR model on the real-sim dataset where the population size parameter $\tau$ is set to $\{1,10,100\}$. All other settings are the same as those in Section "Binary classification based benchmark problems".

**Fig. 2:**
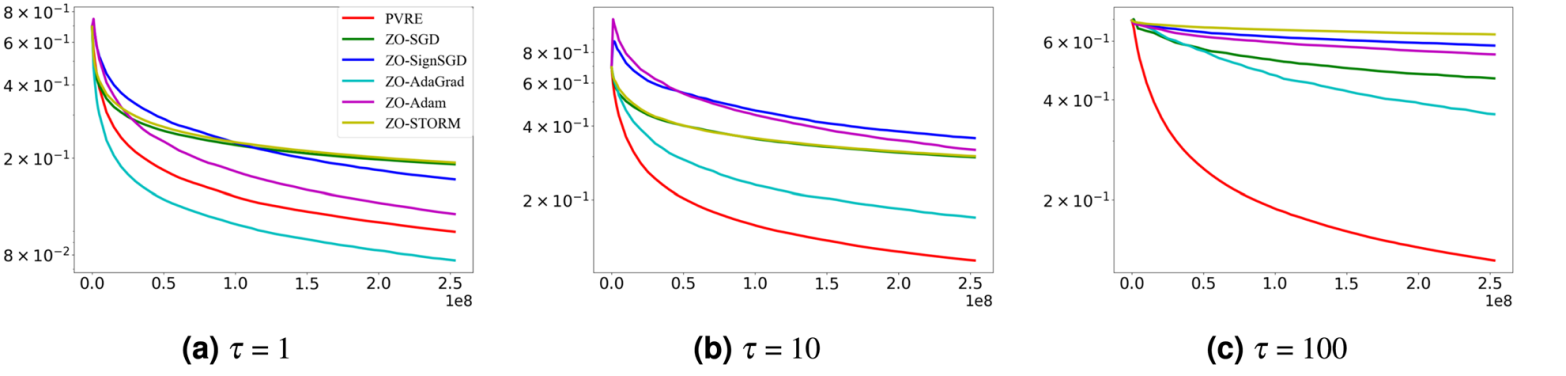
Comparison under different population size settings. The curve displays the training loss versus the number of function evaluations.

Figure 2 shows the experimental results. When the population size is set to 1, the PVRE algorithm essentially degenerates into a strategy similar to a single-point search. In this case, due to the lack of support for population diversity, PVRE cannot effectively use the differences and complementarities within the population to guide the search direction. With the increase in population size, PVRE clearly outperforms the comparison algorithm. It is probably due to that a larger population size increases the diversity of search directions, therefore enhancing exploration of the search space and solution quality.

### Impact of initial step size

Here we examine the influence of the initial step size on the performance of the PVRE algorithm, where the model is LR and the experiments are conducted on the covtype dataset. The initial step size $\gamma _0$ is set to $\{0.01, 0.1, 1\}$, while keeping all other experimental settings identical to those in Section "Binary classification based benchmark problems".

**Fig. 3:**
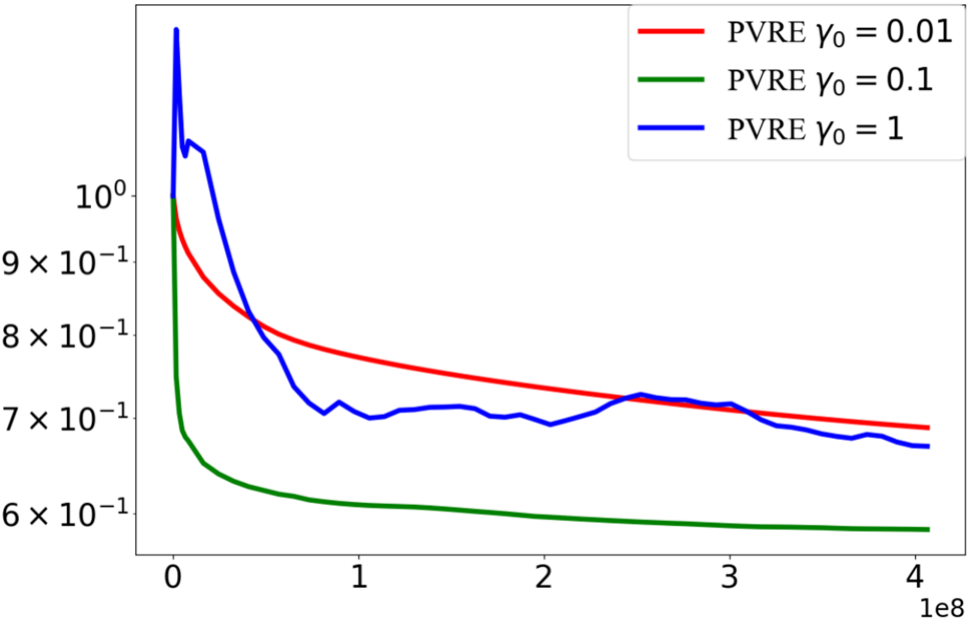
Comparison under different initial step sizes. The curve displays the training loss versus the number of function evaluations.

Figure 3 presents the results on all test instances using performance profiles measured by the loss values. It is evident that the choice of initial step size significantly affects the convergence behavior of PVRE. When $\gamma _0 = 0.01$, the algorithm converges steadily but relatively slowly, resulting in stable yet gradual loss reduction across test instances. Increasing the initial step size to $\gamma _0 = 0.1$ leads to faster initial progress and lower loss values. However, when the initial step size is set to $\gamma _0 = 1$, the loss curve rises initially before decreasing and exhibits noticeable fluctuations throughout the optimization process.

### Black-box adversarial attacks

We consider a real-world task for adversarial perturbation generalization^29^. Given a neural network-based image classifier, the task is to find a perturbation that, when universally applied, degrades the classifier’s accuracy. The data sample $\xi$ in this task consists of an image *z* and its true label *t*. The objective of this task reads:

$$F(x;\xi ) = \underbrace{\max \{0, \log \pi _t(x\oplus z) - \max \limits _{j\ne t} \pi _j(x\oplus z)\}}_{\text {attack loss}} + \underbrace{\frac{\lambda }{2}\Vert x\oplus z-z\Vert ^2}_{\text {distortion}},$$

where $\lambda$ is a regularization coefficient, $x\oplus z$ denotes applying the perturbation *x* to an image *z*, and $\pi _j(x\oplus z)$ is the corresponding probability that the perturbed image is predicted into class *j*. The objective penalizes the top-1 prediction accuracy and therefore encouraging misclassification due to the perturbation. The use of an $\ell _2$ regularization is to prevent the magnitude of the image distortion from growing too fast.

We use the CIFAR-10 dataset^30^ in the experiment and choose 1000 images to build the data distribution. The regularization coefficient $\lambda$ is fixed to 10. The VGG16 model^31^ is used as the image classifier. Suppose this model only receives images in the range $[-0.5,0.5]^n$ where *n* is the dimension of the image space. We can then use the following perturbation operator to make sure the solution space becomes $\mathbb {R}^n$:

$$x\oplus z = \frac{1}{2} \tanh \left( \tanh ^{-1}(2z) + x \right) .$$

The five algorithms considered in the previous experiment are chosen here for comparison. We set $\eta _0$ to $10^{-5}$ and tune $\gamma _0$ in $\{10^{-7},10^{-6},\ldots ,10^{2}\}$. In the data sampling, we use a minibatch of size 5. All other settings are kept the same as in Section "Binary classification based benchmark problems".

**Fig. 4:**
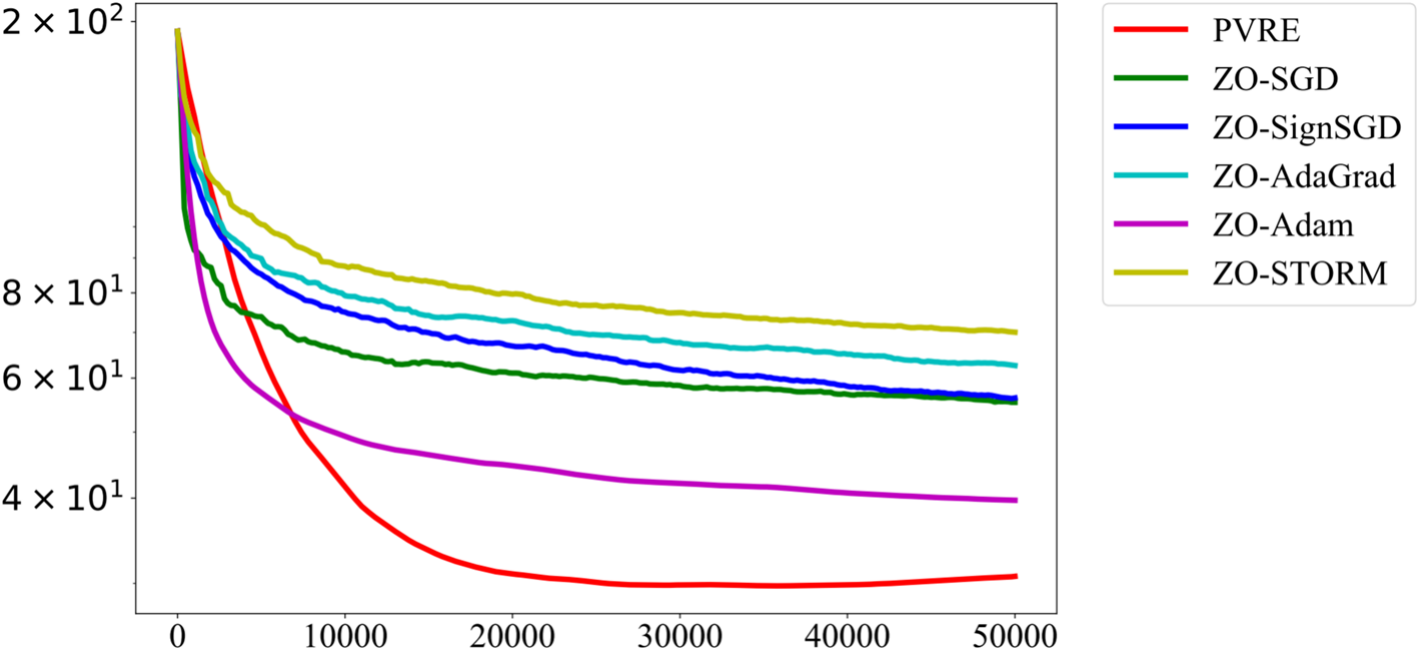
Attack loss versus the number of function evaluations.

**Fig. 5:**
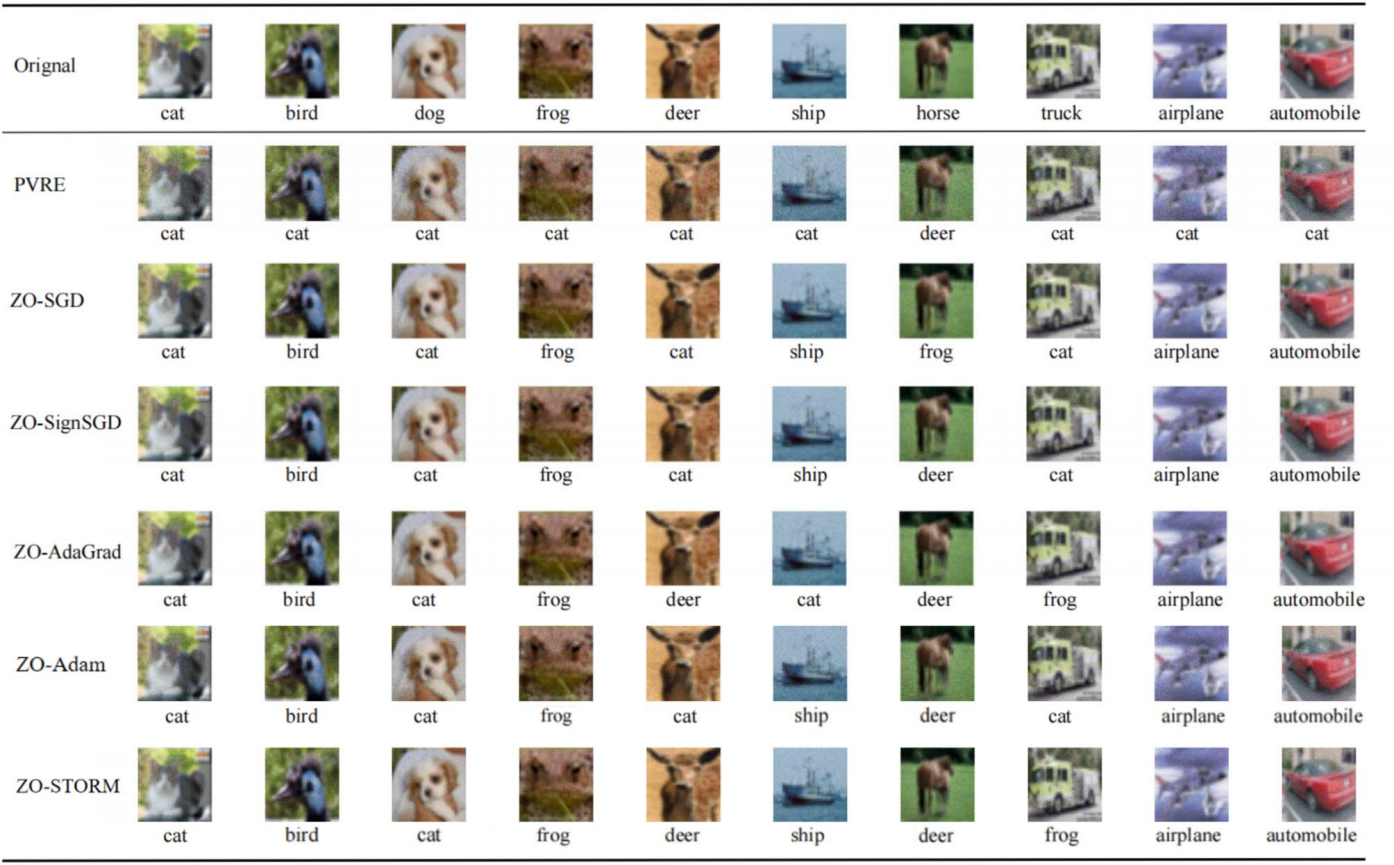
Examples of perturbation images on the CIFAR-10 image dataset. The first row shows the original images and their true labels. The subsequent rows show the perturbed images with the corresponding falsely predicted labels.

Figure 4 shows the trajectory of the attack loss versus the number of function evaluations. It is found that PVRE exhibits the fastest convergence rate among all algorithms. We also present the attack success rate, the final loss, and the averaged distortion (measured by $\frac{1}{N}\sum _{i=1}^N\Vert x\oplus z_i - z_i\Vert ^2$) in Table 2, obtained within a budget of 50000 function evaluations. PVRE obtains the highest attack success rate and the lowest objective value. The perturbation obtained by PVRE exhibits the largest distortion magnitude, but we note that the obtained perturbation is almost imperceptible when applied to the images. In Fig. 5 we present several example images to confirm this. It is found that there is no significant difference between the perturbed images output by different algorithms.

**Table 2:** Final results on universal adversarial perturbation after using a budget of 50000 function evaluations.

Algorithm	Attack success rate	Total loss	Averaged distortion
PVRE	79.60%	30.72	21.20
ZO-SGD	43.10%	55.29	7.40
ZO-SignSGD	44.10%	56.11	7.10
ZO-AdaGrad	39.70%	62.60	6.17
ZO-Adam	58.70%	39.72	10.40
ZO-STORM	33.40%	70.05	5.86

## Related work

First-order stochastic algorithms such as SGD achieve a gradient evaluation complexity of $\mathscr {O}(\epsilon ^{-4})$ when the objective function *f* is smooth^32^. When the component objective *F*, the complexity of zeroth-order methods may align with their first-order counterparts in terms of the $\epsilon$-dependence, but suffer an *n*-dependence slowdown—this being the price paid for not knowing the gradient.

On functions with smooth components, various variance reduction methods exist that improve the complexity of first-order algorithms to $\mathscr {O}(\epsilon ^{-3})$. Remarkable examples include STORM^21^, SVRG^33^, and SPIDER^10^. Extending these methods to zeroth-order settings, however, is not straightforward. For example, it is found that the convergence rate of zeroth-order SVRG methods cannot match the original SVRG in terms of the $\epsilon$-dependence^34,35^. The zeroth-order SPIDER requires periodic gradient evaluations with a megabatch of data samples and a huge population of perturbations along the coordinate directions^10^. The best known result achieved by zeroth-order methods is found in the work^20^ where the authors proved an $n^{3/4}\epsilon ^{-3}$ complexity with a stronger assumption. Our result matches theirs if using the same assumption.

How to achieve adaptability is an active research topic in first-order stochastic optimization. Representative methods such as AdaGrad^27^ and Adam^36^ employ a second-order momentum to perform coordinate-wise normalization, aiming to address the ill-conditioning issue. The STORM method^24^ incorporates an AdaGrad-style momentum rule and simultaneously achieves adaptive convergence and variance reduction. Adam and AdaGrad has also been extended to zeroth-order settings^28,37^, yet they lack employ a variance-reduction mechanism.

EAs in the literature are seldom used for solving stochastic problems, possibly due to the fact that the standard comparison-based evolution operator may yield bias in gradient estimation^38^. We addressed this in a previous work^39^ by using megabatch sampling in distributed settings. It has also been seen that classical evolution gradient search can work well in reinforcement learning and enjoy strong scalability^40^. The main advantage of using EAs is that they typically have better exploration ability on nonconvex landscapes compared to those employing single-solution iterations^41^.

## Conclusion

In this paper, we propose a method called PVRE, which combines Gaussian smoothing and population-based variance reduction techniques. We show that PVRE reaches the objective function complexity of $\tilde{\mathscr {O}}(n\epsilon ^{-3})$ in finding an $\epsilon$-accurate first-order optimum while ensuring the convergence with any initial step-size. PVRE demonstrates promising performance in several experiments on both benchmark problems and real-world applications.

## Supplementary Information

Supplementary Information.

## Data Availability

The datasets used in this study are all publicly available. Specifically, we use four benchmark datasets from the LIBSVM repository—covtype, gisste, rcv1, and real-sim—available at https://www.csie.ntu.edu.tw/~cjlin/libsvmtools/datasets/, as well as the CIFAR-10 dataset, which is accessible from https://www.cs.toronto.edu/~kriz/cifar.html.
