# Supplementary material for: Population-based variance-reduced evolution over stochastic landscapes
Source: Sci Rep. 2025 Oct 7;15:34982. doi: 10.1038/s41598-025-18876-0 (PMC12504688; doi:10.1038/s41598-025-18876-0)
Supplement: Supplementary file 1 — Supplementary Information. [file 41598_2025_18876_MOESM1_ESM.pdf]

Appendix A Technical Lemmas

Lemma A1 (Properties of Gaussian Smoothing)**.** *Assume function $h : \mathbb{R}^n \to \mathbb{R}$ is L-smooth. Then, we have:*

1. *h_η is L-smooth.*
2. *$|h(x) - h_\eta(x)| \leq \frac{\eta^2}{2} Ln, \quad \forall x.$*
3. *$\left\|\nabla h(x) - \nabla h_\eta(x)\right\| \leq \frac{\eta}{2} L(n+3)^{1.5}, \quad \forall x.$*

Proof. The first statement was given in Section 2 of[1]. The second statement was given in Theorem 1 of[1]. The third statement was given in Lemma 3 of[1]. □

Lemma A2 (Boundedness of Gaussian smoothing based gradient estimator)**.** *Assume a function $h : \mathbb{R}^n \to \mathbb{R}$ is L-smooth.*

$$\mathbb{E}_{v\sim\mathcal{N}}[\|g\|^2] \leq \frac{L^2\eta^2}{2}(n+6)^3 + 2(n+4)\|\nabla h(x)\|^2, \quad \forall x. \tag{1}$$

Proof. The unbiasedness property of g is stated in Equation (26) of[1]. Equation 1 is proven as follows:

$$\begin{aligned}
\mathbb{E}[\|g\|^2] &= \mathbb{E}\left[\left\|\frac{h(x+\eta v) - h(x-\eta v)}{2\eta} v\right\|^2\right] \\
&= \frac{1}{4\eta^2}\mathbb{E}\left[|h(x+\eta v) - h(x-\eta v)|^2 \|v\|^2\right] \\
&= \frac{1}{4\eta^2}\mathbb{E}\left[|h(x+\eta v) - h(x-\eta v) - 2\eta\langle\nabla h(x), v\rangle + 2\eta\langle\nabla h(x), v\rangle|^2 \|v\|^2\right] \\
&\leq \frac{1}{2\eta^2}\mathbb{E}\left[|h(x+\eta v) - h(x-\eta v) - 2\eta\langle\nabla h(x), v\rangle|^2 \|v\|^2\right] + \frac{1}{2\eta^2}\mathbb{E}\left[|2\eta\langle\nabla h(x), v\rangle|^2 \|v\|^2\right] \\
&\leq \frac{L^2\eta^2}{2}\mathbb{E}\left[\|v\|^6\right] + 2\mathbb{E}\left[|\langle\nabla h(x), v\rangle|^2 \|v\|^2\right] \\
&\overset{(3)}{\leq} \frac{L^2\eta^2}{2}(n+6)^3 + 2\mathbb{E}\left[|\langle\nabla h(x), v\rangle|^2 \|v\|^2\right] \\
&\overset{(4)}{\leq} \frac{L^2\eta^2}{2}(n+6)^3 + 2(n+4)\|\nabla h(x)\|^2,
\end{aligned}$$

where we used $\|a+b\|^2 \leq 2\|a\|^2 + 2\|b\|^2$ in the first inequality. □

Lemma A3 (Properties on Gaussian vectors)**.** *For n-dimensional vector $v \sim \mathcal{N}$, we have*

$$\mathbb{E}[\|v\|^2] = n, \tag{2}$$

$$\mathbb{E}[\|v\|^p] \leq (n+p)^{\frac{p}{2}}, \quad \forall p > 0, p \neq 1 \tag{3}$$

$$\mathbb{E}[\|\langle v, y\rangle v\|^2] \leq (n+4)\|y\|^2, \quad \forall y \in \mathbb{R}^n. \tag{4}$$

Proof. The first equation is trivial. The second equation is stated in Lemma 1 of[1]. The last equation is stated in Theorem 4 of[1]. □

Lemma A4 (Bounds on the partial sum of divergent p-series)**.** *For all $t \in \mathbb{Z}_+$, we have*

$$\sum_{i=1}^{t} \frac{1}{t} \leq 1 + \log t \tag{5}$$

$$\sum_{i=1}^{t} \frac{1}{t^p} \leq 1 + \frac{t^{1-p} - 1}{1-p}, \forall p \in \mathbb{Z}, p > 1 \tag{6}$$

$$\sum_{i=1}^{t} i^{-\frac{4}{3}}\left(1 - t^{-\frac{2}{3}}\right)^{2(t-i)} \leq 6t^{-\frac{2}{3}} \tag{7}$$

Proof. (5) and (6) were proved in[2]. Here, we prove the last one. Let's start with the following relations:

$$\left(1 - t^{-\frac{2}{3}}\right)^{2(t-i)} \leq t^{-\frac{2}{3}} \Leftrightarrow 2(t-i)\log\left(1 - t^{-\frac{2}{3}}\right) \leq -\frac{2}{3}\log t \Leftrightarrow i \leq t + \frac{\frac{1}{3}\log t}{\log\left(1 - t^{-\frac{2}{3}}\right)}.$$

Using the fact $\log(1+x) \le x, \forall x > -1$, we have $\log\left(1-t^{-\frac{2}{3}}\right) \le -t^{-\frac{2}{3}}$ and therefore

$$i \le \left\lfloor t + \frac{\frac{1}{3}\log t}{-t^{-\frac{2}{3}}} \right\rfloor \le t + \frac{\frac{1}{3}\log t}{\log\left(1-t^{-\frac{2}{3}}\right)} \Rightarrow \left(1-t^{-\frac{2}{3}}\right)^{2(t-i)} \le t^{-\frac{2}{3}}. \tag{8}$$

Now let

$$\mu = t + \frac{\frac{1}{3}\log t}{-t^{-\frac{2}{3}}} = t - \frac{1}{3}t^{\frac{2}{3}}\log t. \tag{9}$$

Then we have

$$\sum_{i=1}^{\lfloor \mu \rfloor} i^{-\frac{4}{3}}\left(1-t^{-\frac{2}{3}}\right)^{2(t-i)} \overset{(8)}{\le} t^{-\frac{2}{3}} \sum_{i=1}^{\lfloor \mu \rfloor} i^{-\frac{4}{3}} \overset{(6)}{\le} 4t^{-\frac{2}{3}}. \tag{10}$$

On the other hand, we can bound $\sum_{i=\lceil \mu \rceil}^{t} i^{-\frac{4}{3}}\left(1-t^{-\frac{2}{3}}\right)^{2(t-i)}$ as

$$\begin{aligned}
\sum_{i=\lceil \mu \rceil}^{t} i^{-\frac{4}{3}}\left(1-t^{-\frac{2}{3}}\right)^{2(t-i)} &\le \lceil \mu \rceil^{-\frac{4}{3}} \sum_{i=\lceil \mu \rceil}^{t} \left(1-t^{-\frac{2}{3}}\right)^{2(t-i)} \le \mu^{-\frac{4}{3}} \sum_{i=0}^{\infty} \left(1-t^{-\frac{2}{3}}\right)^{2i} \\
&= \mu^{-\frac{4}{3}} \frac{1}{1-\left(1-t^{-\frac{2}{3}}\right)^2} \\
&\le \mu^{-\frac{4}{3}} \frac{1}{1-\left(1-t^{-\frac{2}{3}}\right)} \\
&= \mu^{-\frac{4}{3}} t^{\frac{2}{3}} \\
&\overset{(9)}{=} \left(t - \frac{1}{3}t^{\frac{2}{3}}\log t\right)^{-\frac{4}{3}} t^{\frac{2}{3}} \\
&= t^{-\frac{2}{9}} \left(t^{\frac{1}{3}} - \frac{1}{3}\log t\right)^{-\frac{4}{3}} \\
&\overset{(*)}{\le} t^{-\frac{2}{9}} \left(2^{-\frac{3}{4}} t^{\frac{1}{3}}\right)^{-\frac{4}{3}} \\
&\le 2t^{-\frac{2}{3}}
\end{aligned}$$

where $(*)$ is due to $\left(1-2^{-\frac{3}{4}}\right)t^{\frac{1}{3}} \ge \frac{1}{3}\log t$ for all $t \ge 1$. Putting this and (10) together, we obtain the final bound in (7). □

Appendix B Proofs of Lemmas 3.1 to 3.4

Proof of Lemma 3.1. According to Lemma A1 and the smoothness assumption of f, we know the Gaussian smoothing f_{η_t} is L-smooth. Then, using Lemma 2 of[3], for iterations of the form $x_{t+1} = x_t - \gamma_t \frac{d_t}{\|d_t\|}$, we have

$$f_{\eta_t}(x_{t+1}) \le f_{\eta_t}(x_t) - \frac{\gamma_t}{3}\|\nabla f_{\eta_t}(x_t)\| + \frac{8}{3}\gamma_t\|\varepsilon_t\| + \frac{L}{2}\gamma_t^2. \tag{11}$$

The difference between $f_{\eta_t}(x_{t+1})$ and $f_{\eta_{t+1}}(x_{t+1})$ can be bounded as

$$\begin{aligned}
|f_{\eta_{t+1}}(x_{t+1}) - f_{\eta_t}(x_{t+1})| &= |\mathbb{E}[f(x_{t+1}+\eta_{t+1}v) - f(x_{t+1}+\eta_t v)]| \\
&= |\mathbb{E}[f(x_{t+1}+\eta_{t+1}v) - f(x_{t+1}) - \eta_{t+1}\langle \nabla f(x_{t+1}), v\rangle] \\
&\quad - \mathbb{E}[f(x_{t+1}+\eta_t v) - f(x_{t+1}) - \eta_t\langle \nabla f(x_{t+1}), v\rangle]| \\
&\le \mathbb{E}[|f(x_{t+1}+\eta_{t+1}v) - f(x_{t+1}) - \eta_{t+1}\langle \nabla f(x_{t+1}), v\rangle|] \\
&\quad + \mathbb{E}[|f(x_{t+1}+\eta_t v) - f(x_{t+1}) - \eta_t\langle \nabla f(x_{t+1}), v\rangle|]
\end{aligned}$$

where the first equality is by definition, the second equality is due to $\mathbb{E}[v] = 0$ for $v \sim \mathcal{N}$, and in the inequality we used Jensen's inequality. Using the smoothness assumption of f and a standard quadratic bound, we further have

$$|f_{\eta_{t+1}}(x_{t+1}) - f_{\eta_t}(x_t)| \le \frac{L}{2}(\eta_{t+1}^2 + \eta_t^2)\mathbb{E}[\|v\|^2] = \frac{L}{2}(\eta_{t+1}^2 + \eta_t^2)n$$

where the second inequality is due to (2). Combing this with (11) completes the proof. □

Proof of Lemma 3.2. By construction, we have

$$\mathbb{E}\left[\|g_t - \nabla f_{\eta_t}(x_t)\|^2\right] = \mathbb{E}\left[\left\|\frac{1}{\tau}\sum_{k=1}^{\tau} g_{t,k} - \nabla f_{\eta_t}(x_t)\right\|^2\right]$$

Since $g_{t,k}$ is an unbiased estimation of $\nabla f_{\eta_t}(x_t)$, we have

$$\begin{aligned}\mathbb{E}\left[\|g_t - \nabla f_{\eta_t}(x_t)\|^2\right] &= \frac{1}{\tau^2}\sum_{k=1}^{\tau}\mathbb{E}\left[\|g_{t,k} - \nabla f_{\eta_t}(x_t)\|^2\right] \\ &\le \frac{1}{\tau^2}\sum_{k=1}^{\tau}\mathbb{E}\left[\|g_{t,k}\|^2\right] \\ &\le \frac{1}{\tau^2}\left(\frac{L^2}{2}\eta_t^2(n+6)^3 + 2(n+4)\mathbb{E}[\|\nabla F(x_t;\xi_t)\|^2]\right)\end{aligned} \tag{12}$$

where the equality follows from the unbiasedness property of $g_{t,k}$, the first inequality is due to $\mathbb{E}\left[\|a - \mathbb{E}[a]\|^2\right] \le \mathbb{E}\left[\|a\|^2\right]$, and the second inequality is due to a bound established in the appendix.

By the assumptions of unbiased and bounded sampling and the boundedness of the gradient, we have

$$\mathbb{E}[\|\nabla F(x_t;\xi_t)\|^2] = \|\nabla f(x_t)\|^2 + \mathbb{E}[\|\nabla f(x_t) - \nabla F(x_t;\xi_t)\|^2] \le G^2 + \sigma^2.$$

Plugging this into (12) gives the desired bound. □

Proof of Lemma 3.3.

$$\begin{aligned} g_{t,i} - \tilde{g}_{t-1,i} &= \frac{F(x_t + \eta_t v_{t,i};\xi_{t,i}) - F(x_t - \eta_t v_{t,i};\xi_{t,i})}{2\eta_t} v_{t,i} \\ &\quad - \frac{F(x_{t-1} + \eta_{t-1} v_{t,i};\xi_{t,i}) - F(x_{t-1} - \eta_{t-1} v_{t,i};\xi_{t,i})}{2\eta_{t-1}} v_{t,i} \\ &= \frac{F(x_t + \eta_t v_{t,i};\xi_{t,i}) - F(x_t - \eta_t v_{t,i};\xi_{t,i}) - 2\eta_t \langle \nabla F(x_t;\xi_{t,i}), v_{t,i}\rangle}{2\eta_t} v_{t,i} \\ &\quad - \frac{F(x_{t-1} + \eta_{t-1} v_{t,i};\xi_{t,i}) - F(x_{t-1} - \eta_{t-1} v_{t,i};\xi_{t,i}) - 2\eta_{t-1}\langle \nabla F(x_{t-1};\xi_{t,i}), v_{t,i}\rangle}{2\eta_{t-1}} v_{t,i} \\ &\quad + \langle \nabla F(x_t;\xi_{t,i}) - \nabla F(x_{t-1};\xi_{t,i}), v_{t,i}\rangle. \end{aligned}$$

Using the fact $\|a+b+c\|^2 \le 3\|a\|^2 + 3\|b\|^2 + 3\|c\|^2$, we obtain

$$\begin{aligned} &\|g_{t,i} - \tilde{g}_{t-1,i}\|^2 \\ &\le \frac{3|F(x_t + \eta_t v_{t,i};\xi_t) - F(x_t - \eta_t v_{t,i};\xi_t) - 2\eta_t\langle \nabla F(x_t;\xi_t), v_{t,i}\rangle|^2}{4\eta_t^2}\|v_{t,i}\|^2 \\ &\quad + \frac{3|F(x_{t-1} + \eta_{t-1} v_{t,i};\xi_t) - F(x_{t-1} - \eta_{t-1} v_{t,i};\xi_t) - 2\eta_{t-1}\langle \nabla F(x_{t-1};\xi_t), v_{t,i}\rangle|^2}{4\eta_{t-1}^2}\|v_{t,i}\|^2 \\ &\quad + 3|\langle \nabla F(x_t;\xi_t) - \nabla F(x_{t-1};\xi_t), v_{t,i}\rangle|^2. \end{aligned}$$

By the smoothness assumption of F, and using the bound $|h(x+v)-h(x-v)-2\langle\nabla h(x),v\rangle| \leq L\|v\|^2$, we can bound the first two terms as

$$\begin{aligned}
\mathbb{E}\left[\|g_{t,i}-\tilde{g}_{t-1,i}\|^2\right] &\leq \frac{3L^2(\eta_t^2+\eta_{t-1}^2)}{4}\mathbb{E}\left[\|v_{t,i}\|^6\right]+3\mathbb{E}\left[\|\left\langle\nabla F(x_t;\xi_t)-\nabla F(x_{t-1};\xi_t),v_{t,i}\right\rangle\|^2\right] \\
&\overset{(4)}{\leq} \frac{3L^2(\eta_t^2+\eta_{t-1}^2)}{4}\mathbb{E}\left[\|v_{t,i}\|^6\right]+3(n+4)\mathbb{E}\left[\|\nabla F(x_t;\xi_t)-\nabla F(x_{t-1};\xi_t)\|^2\right] \\
&\overset{(3)}{\leq} \frac{3L^2(\eta_t^2+\eta_{t-1}^2)}{4}(n+6)^3+3(n+4)\mathbb{E}\left[\|\nabla F(x_t;\xi_t)-\nabla F(x_{t-1};\xi_t)\|^2\right] \\
&\leq \frac{3L^2(\eta_t^2+\eta_{t-1}^2)}{4}(n+6)^3+3(n+4)L^2\mathbb{E}\left[\|x_t-x_{t-1}\|^2\right].
\end{aligned}$$

Using the update rule $x_{t+1}=x_t-\gamma_t\frac{d_t}{\|d_t\|}$ completes the proof.

□

Proof of Lemma 3.4. Rewrite the gradient estimation error at iteration $t+1$ as

$$\begin{aligned}
\varepsilon_{t+1} &= d_{t+1}-\nabla f_{\eta_{t+1}}(x_{t+1}) \\
&= g_{t+1}+(1-a_t)(d_t-\tilde{g}_t)-\nabla f_{\eta_{t+1}}(x_{t+1}) \\
&= (1-a_t)d_t+a_t g_{t+1}+(1-a_t)(g_{t+1}-\tilde{g}_t)-\nabla f_{\eta_{t+1}}(x_{t+1}) \\
&= (1-a_t)\varepsilon_t+(1-a_t)\nabla f_{\eta_t}(x_t)+a_t g_{t+1}+(1-a_t)(g_{t+1}-\tilde{g}_t)-\nabla f_{\eta_{t+1}}(x_{t+1}) \\
&= (1-a_t)\varepsilon_t+(1-a_t)\left(\nabla f_{\eta_t}(x_t)-\nabla f_{\eta_{t+1}}(x_{t+1})\right)+a_t(g_{t+1}-\nabla f_{\eta_{t+1}}(x_{t+1}))+(1-a_t)(g_{t+1}-\tilde{g}_t) \\
&= (1-a_t)\varepsilon_t+a_t(g_{t+1}-\nabla f_{\eta_{t+1}}(x_{t+1}))+(1-a_t)\left(g_{t+1}-\tilde{g}_t+\nabla f_{\eta_t}(x_t)-\nabla f_{\eta_{t+1}}(x_{t+1})\right).
\end{aligned}$$

Introduce the following notations for convenience:

$$\begin{cases}
\delta_{t+1} &= g_{t+1}-\nabla f_{\eta_{t+1}}(x_{t+1}) \\
\psi_{t+1} &= g_{t+1}-\tilde{g}_t+\nabla f_{\eta_t}(x_t)-\nabla f_{\eta_{t+1}}(x_{t+1}) \\
u_{t+1} &= a_t\delta_{t+1}+(1-a_t)\psi_{t+1}
\end{cases}.$$

With these notations, we unroll ε_{t+1} as

$$\begin{aligned}
\varepsilon_{t+1} &= (1-a_t)\varepsilon_t+u_{t+1} \\
&= (1-a_t)\left((1-a_{t-1})\varepsilon_{t-1}+u_t\right)+u_{t+1} \\
&= \cdots \\
&= \varepsilon_0\prod_{i=0}^{t}(1-a_i)+\sum_{i=1}^{t+1}u_i\prod_{j=i}^{t}(1-a_j) \\
&= \varepsilon_0\prod_{i=0}^{t}(1-a_i)+\sum_{i=1}^{t+1}a_{i-1}\delta_i\prod_{j=i}^{t}(1-a_j)+\sum_{i=1}^{t+1}\psi_i\prod_{j=i-1}^{t}(1-a_j),
\end{aligned}$$

and therefore

$$\|\varepsilon_t\| \leq \prod_{i=0}^{t-1}(1-a_i)\|\varepsilon_0\|+\left\|\sum_{i=1}^{t}a_{i-1}\delta_i\prod_{j=i}^{t-1}(1-a_j)\right\|+\left\|\sum_{i=1}^{t}\psi_i\prod_{j=i-1}^{t-1}(1-a_j)\right\|.$$

Now we bound the accumulated gradient estimation errors over iterations, weighted by the step-sizes, as

$$\begin{aligned}
\sum_{t=0}^{T-1}\gamma_t\mathbb{E}[\|\varepsilon_t\|] \leq \underbrace{\gamma_0\mathbb{E}[\|\varepsilon_0\|]+\sum_{t=1}^{T-1}\gamma_t\prod_{i=0}^{t-1}(1-a_i)\mathbb{E}[\|\varepsilon_0\|]}_{\mathfrak{A}} \\
+\underbrace{\sum_{t=1}^{T-1}\gamma_t\mathbb{E}\left[\left\|\sum_{i=1}^{t}a_{i-1}\delta_i\prod_{j=i}^{t-1}(1-a_j)\right\|\right]}_{\mathfrak{B}}+\underbrace{\sum_{t=1}^{T-1}\gamma_t\mathbb{E}\left[\left\|\sum_{i=1}^{t}\psi_i\prod_{j=i-1}^{t-1}(1-a_j)\right\|\right]}_{\mathfrak{C}}.
\end{aligned} \tag{13}$$

Term $\mathfrak{A}$ of (13) can be bounded as

$$
\begin{aligned}
\mathfrak{A} &\leq \mathbb{E}[\|\varepsilon_0\|]\left(\gamma_0 + \sum_{t=1}^{T-1} \gamma_t (1-a_{t-1})^t\right) \\
&= \mathbb{E}[\|\varepsilon_0\|]\gamma_0\left(1 + \sum_{t=1}^{T-1} (1+t)^{-\frac{2}{3}} \left(1 - t^{-\frac{2}{3}}\right)^t\right) \\
&\leq \mathbb{E}[\|\varepsilon_0\|]\gamma_0\left(1 + \sum_{t=1}^{T-1} (1+t)^{-\frac{2}{3}} \frac{1}{1+t^{\frac{1}{3}}}\right) \\
&\leq \mathbb{E}[\|\varepsilon_0\|]\gamma_0\left(1 + \sum_{t=1}^{T-1} (1+t)^{-1}\right) \\
&\overset{(5)}{\leq} \mathbb{E}[\|\varepsilon_0\|]\gamma_0(1+\log T)
\end{aligned}
$$

where the first inequality is due to the decreasing nature of a_i, the second inequality is due to $(1+x)^r \leq \frac{1}{1-rx}$ for $x \in [-1, 0]$, and the third inequality is due to $1+t^{\frac{1}{3}} \geq (1+t)^{\frac{1}{3}}$. The initial error ε_0 can be bounded as

$$
\mathbb{E}[\|\varepsilon_0\|^2] = \mathbb{E}[\|d_0 - \nabla f_{\eta_0}(x_0)\|^2] = \mathbb{E}[\|g_0 - \nabla f_{\eta_0}(x_0)\|^2] \leq \frac{\rho_0}{\tau}
$$

where the inequality follows from Lemma 3.2. Then, using Jensen's inequality, we obtain

$$
\mathfrak{A} \leq \mathbb{E}[\|\varepsilon_0\|]\gamma_0(1+\log T) \leq \sqrt{\mathbb{E}[\|\varepsilon_0\|^2]}\gamma_0(1+\log T) \leq \gamma_0\sqrt{\frac{\rho_0}{\tau}}(1+\log T). \tag{14}
$$

Term $\mathfrak{B}$ of (13) can be bounded as

$$
\mathfrak{B} \leq \sum_{t=1}^{T-1} \gamma_t \sqrt{\mathbb{E}\left[\left\|\sum_{i=1}^{t} a_{i-1}\delta_i \prod_{j=i}^{t-1}(1-a_j)\right\|^2\right]} = \sum_{t=1}^{T-1} \gamma_t \sqrt{\sum_{i=1}^{t} a_{i-1}^2 \mathbb{E}\left[\|\delta_i\|^2\right] \prod_{j=i}^{t-1}(1-a_j)^2} \tag{15}
$$

where the inequality is due to Jensen's inequality, and the equality is due to that δ_i has zero mean, i.e.,

$$
\mathbb{E}[\delta_i] = \mathbb{E}[g_i - \nabla f_{\eta_i}(x_i)] = \frac{1}{\tau}\sum_{k=1}^{\tau} \mathbb{E}_{\xi_{i,k}\sim\mathscr{D}}[\mathbb{E}_{v_{i,k}\sim\mathscr{N}}[g_{i,k}] - \nabla F_{\eta_i}(x_i;\xi_{i,k})] = 0.
$$

Here, the last equality stems from the property of Gaussian smoothing. Now we proceed with bounding $\mathbb{E}\left[\|\delta_i\|^2\right]$ as

$$
\begin{aligned}
\mathbb{E}\left[\|\delta_i\|^2\right] &= \mathbb{E}\left[\|g_i - \nabla f_{\eta_i}(x_i)\|^2\right] \\
&= \mathbb{E}\left[\left\|\frac{1}{\tau}\sum_{k=1}^{\tau} g_{i,k} - \nabla f_{\eta_i}(x_i)\right\|^2\right] \\
&= \frac{1}{\tau^2}\sum_{k=1}^{\tau} \mathbb{E}\left[\|g_{i,k} - \nabla f_{\eta_i}(x_i)\|^2\right] \\
&\leq \frac{\rho_i}{\tau}
\end{aligned}
$$

where in the last equality we used the unbiasedness property of $g_{i,k}$ again, and the inequality is due to Lemma 3.2. Plugging

this into (15) gives

$$
\begin{aligned}
\mathfrak{B} &\le \sqrt{\frac{\rho_0}{\tau}} \sum_{t=1}^{T-1} \gamma_t \sqrt{\sum_{i=1}^{t} a_{i-1}^2 \prod_{j=i}^{t-1} (1-a_j)^2} \\
&\le \sqrt{\frac{\rho_0}{\tau}} \sum_{t=1}^{T-1} \gamma_t \sqrt{\sum_{i=1}^{t} a_{i-1}^2 (1-a_{t-1})^{2(t-i)}} \\
&= \sqrt{\frac{\rho_0}{\tau}} \sum_{t=1}^{T-1} \gamma_0 (t+1)^{-\frac{2}{3}} \sqrt{\sum_{i=1}^{t} i^{-\frac{4}{3}} \left(1-t^{-\frac{2}{3}}\right)^{2(t-i)}} \\
&\overset{(7)}{\le} \sqrt{\frac{\rho_0}{\tau}} \sum_{t=1}^{T-1} \gamma_0 (t+1)^{-\frac{2}{3}} \sqrt{6t^{-\frac{2}{3}}} \\
&\le 3\sqrt{\frac{\rho_0}{\tau}} \sum_{t=1}^{T-1} \frac{\gamma_0}{t} \\
&\overset{(5)}{\le} 3\sqrt{\frac{\rho_0}{\tau}} \gamma_0 (1+\log T),
\end{aligned}
\tag{16}
$$

where the first two inequalities use the decreasing nature of ρ_t and α_t.

Term $\mathfrak{C}$ of (13), again, can be bounded using Jensen's inequality as

$$
\mathfrak{C} \le \sum_{t=1}^{T-1} \gamma_t \sqrt{\mathbb{E}\left[\left\| \sum_{i=1}^{t} \psi_i \prod_{j=i-1}^{t-1} (1-a_j) \right\|^2\right]}. \tag{17}
$$

Since g_i and $\tilde{g}_{i-1}$ are unbiased estimations of $\nabla f_{\eta_i}(x_i)$ and $\nabla f_{\eta_{i-1}}(x_i)$, respectively, we have $\mathbb{E}[\psi_i] = 0$, which means

$$
\text{the r.h.s. of (17)} = \sum_{t=1}^{T-1} \gamma_t \sqrt{\sum_{i=1}^{t} \mathbb{E}\left[\|\psi_i\|^2\right] \prod_{j=i-1}^{t-1} (1-a_j)^2}. \tag{18}
$$

We can bound ψ_i as

$$
\begin{aligned}
\mathbb{E}[\|\psi_i\|^2] &= \mathbb{E}\left[\left\|g_i - \tilde{g}_{i-1} + \nabla f_{\eta_{i-1}}(x_{i-1}) - \nabla f_{\eta_i}(x_i)\right\|^2\right] \\
&= \frac{1}{\tau^2} \sum_{k=1}^{\tau} \mathbb{E}\left[\left\|g_{i,k} - \tilde{g}_{i-1,k} + \nabla f_{\eta_{i-1}}(x_{i-1}) - \nabla f_{\eta_i}(x_i)\right\|^2\right] \\
&\le \frac{1}{\tau^2} \sum_{k=1}^{\tau} \mathbb{E}\left[\|g_{i,k} - \tilde{g}_{i-1,k}\|^2\right] \\
&\le \frac{3L^2\eta_{i-1}^2}{2\tau}(n+6)^3 + \frac{3L^2\gamma_{i-1}^2}{\tau}(n+4) \\
&\le \frac{9L^2\gamma_{i-1}^2}{2\tau}(n+6)
\end{aligned}
$$

where the second equality is due to the unbiasedness properties of $g_{i,k}$ and $\tilde{g}_{i-1,k}$, the first inequality is due to $\mathbb{E}\left[\|a - \mathbb{E}[a]\|^2\right] \le$

$\mathbb{E}[\|a\|^2]$, and the second inequality is due to Lemma 3.3 and $\eta_i \le \eta_{i-1}$. Now plugging the above into (18), we obtain

$$\begin{aligned}
\mathfrak{C} &\le \sum_{t=1}^{T-1} \gamma_t \sqrt{\sum_{i=1}^{t} \frac{9L^2\gamma_{i-1}^2}{2\tau}(n+6) \prod_{j=i-1}^{t-1}(1-a_j)^2} \\
&= L\sqrt{\frac{n+6}{\tau}} \sum_{t=1}^{T-1} \gamma_t \sqrt{\frac{9}{2}\sum_{i=1}^{t} \gamma_{i-1}^2 \prod_{j=i-1}^{t-1}(1-a_j)^2} \\
&\le L\sqrt{\frac{n+6}{\tau}} \sum_{t=1}^{T-1} \gamma_t \sqrt{\frac{9}{2}\sum_{i=1}^{t} \gamma_{i-1}^2 (1-a_{t-1})^{2(t-i)}} \\
&\le \gamma_0^2 L\sqrt{\frac{n+6}{\tau}} \sum_{t=1}^{T-1} t^{-\frac{2}{3}} \sqrt{\frac{9}{2}\sum_{i=1}^{t} i^{-\frac{4}{3}} \left(1-t^{-\frac{2}{3}}\right)^{2(t-i)}} \\
&\overset{(7)}{\le} \gamma_0^2 L\sqrt{27\frac{n+6}{\tau}} \sum_{t=1}^{T-1} \frac{1}{t} \\
&\overset{(5)}{\le} 6\gamma_0^2 L\sqrt{\frac{n+6}{\tau}}(1+\log T),
\end{aligned}$$

where the second inequality uses the decreasing property of a_j and the third inequality uses the facts $a_{t-1} = i^{-\frac{2}{3}}$ and $\gamma_t \le \gamma_{t-1} = \gamma_0 t^{-\frac{2}{3}}$. Plugging into (13) the above bound as well as those in (14) and (16), one finally reaches

$$\sum_{t=0}^{T-1} \gamma_t \mathbb{E}[\|\varepsilon_t\|] \le 4\gamma_0 \sqrt{\frac{\rho_0}{\tau}}(1+\log T) + 6\gamma_0^2 L\sqrt{\frac{n+6}{\tau}}(1+\log T). \tag{19}$$

Here the term ρ_0, by its definition given in Lemma 3.2, can be bounded as

$$\begin{aligned}
\rho_0 &= \frac{L^2}{2}\eta_0^2(n+6)^3 + 2(n+4)(G^2+\sigma^2) \\
&= \frac{L^2}{2}\gamma_0^2(n+6) + 2(n+4)(G^2+\sigma^2) \\
&\le (n+6)\left(\frac{L^2}{2}\gamma_0^2 + 2(G^2+\sigma^2)\right).
\end{aligned}$$

Plugging the above into (19) gives

$$\begin{aligned}
\sum_{t=0}^{T-1} \gamma_t \mathbb{E}[\|\varepsilon_t\|] &\le \left(4\gamma_0\sqrt{\frac{\rho_0}{\tau}} + 6\gamma_0^2 L\sqrt{\frac{n+6}{\tau}}\right)(1+\log T) \\
&\le \left(4\gamma_0\sqrt{\frac{L^2}{2}\gamma_0^2 + 2(G^2+\sigma^2)} + 6\gamma_0^2 L\right)(1+\log T)\sqrt{\frac{n+6}{\tau}} \\
&\le \left(4\gamma_0\left(\sqrt{\frac{L^2}{2}\gamma_0^2} + \sqrt{2(G^2+\sigma^2)}\right) + 6\gamma_0^2 L\right)(1+\log T)\sqrt{\frac{n+6}{\tau}} \\
&\le \left(6\gamma_0(G+\sigma) + 9\gamma_0^2 L\right)(1+\log T)\sqrt{\frac{n+6}{\tau}} \\
&\le 9\gamma_0\left(G+\sigma+\gamma_0 L\right)(1+\log T)\sqrt{\frac{n+6}{\tau}}.
\end{aligned} \tag{20}$$

The proof the completes. □

Appendix C Proof of Theorem 1

Proof. By Lemma 3.1 and the setting $\eta_{t+1} < \eta_t = \frac{\gamma_t}{\sqrt{n}}$, we have

$$f_{\eta_{t+1}}(x_{t+1}) \le f_{\eta_t}(x_t) - \frac{\gamma_t}{3}\|\nabla f_{\eta_t}(x_t)\| + \frac{8}{3}\gamma_t\|\varepsilon_t\| + \frac{3}{2}L\gamma_t^2.$$

Taking total expectation at both sides and summing over $t = 0, \dots, T-1$, we obtain

$$\mathbb{E}[f_{\eta_T}(x_T)] \leq f_{\eta_0}(x_0) - \frac{1}{3}\sum_{t=0}^{T-1}\gamma_t\mathbb{E}[\|\nabla f_{\eta_t}(x_t)\|] + \frac{8}{3}\sum_{t=0}^{T-1}\gamma_t\mathbb{E}[\|\varepsilon_t\|] + \frac{3}{2}L\sum_{t=0}^{T-1}\gamma_t^2.$$

Using the smoothness assumption of f and Lemma A1, we have

$$f_{\eta_0}(x_0) \leq f(x_0) + \frac{L}{2}\eta_0^2 n \quad \text{and} \quad f_{\eta_T}(x_T) \geq f(x_T) - \frac{L}{2}\eta_T^2 n$$

and therefore

$$\frac{1}{3}\sum_{t=0}^{T-1}\mathbb{E}[\gamma_t\|\nabla f_{\eta_t}(x_t)\|] \leq \Delta_f + L\gamma_0^2 + \frac{8}{3}\sum_{t=0}^{T-1}\gamma_t\mathbb{E}[\|\varepsilon_t\|] + \frac{3}{2}L\sum_{t=0}^{T-1}\gamma_t^2, \tag{21}$$

where we used the facts $f(x_T) \geq f_*$ and $\eta_T \leq \eta_0 \leq \frac{\gamma_0}{\sqrt{n}}$. Note that, with the setting $\gamma_t = \gamma_0(1+t)^{-\frac{2}{3}}$, the l.h.s. of (21) can be bounded from below as

$$\frac{1}{3}\sum_{t=0}^{T-1}\gamma_t\mathbb{E}[\|\nabla f_{\eta_t}(x_t)\|] = \frac{\gamma_0}{3}\sum_{t=0}^{T-1}(t+1)^{-\frac{2}{3}}\mathbb{E}[\|\nabla f_{\eta_t}(x_t)\|] \geq \frac{\gamma_0}{3}T^{\frac{1}{3}}\sum_{t=0}^{T-1}\mathbb{E}[\|\nabla f_{\eta_t}(x_t)\|].$$

In addition, with the setting $\gamma_t = \gamma_0(1+t)^{-\frac{2}{3}}$, the last term of the r.h.s. of (21) can be upper bounded by

$$\frac{3}{2}L\sum_{t=0}^{T-1}\gamma_t^2 = \frac{3\gamma_0^2}{2}L\sum_{t=0}^{T-1}(t+1)^{-\frac{4}{3}} \overset{(6)}{\leq} 6\gamma_0^2 L.$$

Plugging these bounds into (21) and rearranging the terms gives

$$\frac{1}{T}\sum_{t=0}^{T-1}\mathbb{E}[\|\nabla f_{\eta_t}(x_t)\|] \leq T^{-\frac{1}{3}}\left(\frac{3\Delta_f}{\gamma_0} + \frac{8}{\gamma_0}\sum_{t=0}^{T-1}\gamma_t\mathbb{E}[\|\varepsilon_t\|] + 21\gamma_0 L\right). \tag{22}$$

Now applying the bound in Lemma 3.4, we obtain

$$\begin{aligned}
\frac{1}{T}\sum_{t=0}^{T-1}\mathbb{E}[\|\nabla f_{\eta_t}(x_t)\|] &\leq T^{-\frac{1}{3}}\left(\frac{3\Delta_f}{\gamma_0} + \frac{8}{\gamma_0}\sum_{t=0}^{T-1}\gamma_t\mathbb{E}[\|\varepsilon_t\|] + 21\gamma_0 L\right) \\
&\leq T^{-\frac{1}{3}}\left(\frac{3\Delta_f}{\gamma_0} + 72(\gamma_0 L + G + \sigma)(1+\log T)\sqrt{\frac{n+6}{\tau}} + 21\gamma_0 L\right) \qquad (23) \\
&\leq 72T^{-\frac{1}{3}}\left(\frac{\Delta_f}{\gamma_0} + (\gamma_0 L + G + \sigma)(1+\log T)\sqrt{\frac{n+6}{\tau}} + \gamma_0 L\right).
\end{aligned}$$

According to Lemma A1, we have

$$\left\|\nabla f_{\eta_t}(x_t) - \nabla f(x_t)\right\| \leq \frac{\eta_t}{2}L(n+3)^{1.5},$$

and it follows that

$$\begin{aligned}
\frac{1}{T}\sum_{t=0}^{T-1}\mathbb{E}[\|\nabla f(x_t)\|] &\leq \frac{1}{T}\sum_{t=0}^{T-1}\mathbb{E}[\|\nabla f_{\eta_t}(x_t)\|] + \frac{1}{T}\sum_{t=0}^{T-1}\mathbb{E}[\|\nabla f_{\eta_t}(x_t) - \nabla f(x_t)\|] \\
&\leq \frac{1}{T}\sum_{t=0}^{T-1}\mathbb{E}[\|\nabla f_{\eta_t}(x_t)\|] + \frac{L}{2}(n+3)^{1.5}\frac{1}{T}\sum_{t=0}^{T-1}\eta_t \\
&\leq \frac{1}{T}\sum_{t=0}^{T-1}\mathbb{E}[\|\nabla f_{\eta_t}(x_t)\|] + \frac{L(n+3)^{1.5}}{2(n+6)}\gamma_0\frac{1}{T}\sum_{t=0}^{T-1}(t+1)^{-\frac{2}{3}} \\
&\overset{(6)}{\leq} \frac{1}{T}\sum_{t=0}^{T-1}\mathbb{E}[\|\nabla f_{\eta_t}(x_t)\|] + 2L\sqrt{n+6}\gamma_0 T^{-\frac{2}{3}}.
\end{aligned}$$

Plugging (23) into above completes the proof. □

References

1. Nesterov, Y. & Spokoiny, V. Random Gradient-Free Minimization of Convex Functions. *Foundations Comput. Math.* **17**, 527–566 (2017).

2. Chlebus, E. An approximate formula for a partial sum of the divergent p-series. *Appl. Math. Lett.* **22**, 732–737, DOI: 10.1016/j.aml.2008.07.007 (2009).

3. Cutkosky, A. & Mehta, H. Momentum improves normalized sgd. In *International Conference on Machine Learning*, 2260–2268 (PMLR, 2020).
